# Joint trajectories of health behaviors and their associations with healthcare utilization and expenditure

**DOI:** 10.3389/ijph.2026.1609686

**Published:** 2026-09-25

**Authors:** Jeong Min Yang, Jang Mook Kim, Tae Su Jang, Jieun Hwang

**Affiliations:** 1 National Infectious Disease Hospital Establishment Task Force, National Medical Center, Seoul, Republic of Korea; 2 Department of Health Administration, College of Health Science, Dankook University, Cheonan-si, Republic of Korea

**Keywords:** body mass index, drinking, health behavior, healthcare service, physical activity

## Abstract

**Objectives:**

This study identified sex-specific trajectories of key health behaviors among middle-aged and older adults and examined their longitudinal associations with healthcare utilization and expenditure.

**Methods:**

We analyzed 167,343 Korean adults aged 40–79 years from 2002 to 2019 using the National Health Insurance Service Health Screening Cohort. Group-based multi-trajectory modeling identified joint trajectories of smoking, alcohol consumption, physical activity, and BMI. Associations with healthcare outcomes were assessed using generalized estimating equations stratified by sex and age group.

**Results:**

Six distinct health behavior trajectories were identified in both sexes. Among men, the higher moderate-risk trajectory showed fewer outpatient visits but longer hospital stays. Among women, the higher severe-risk trajectory was associated with increased expenditures and higher healthcare utilization. Age-stratified analyses revealed stronger associations for severe-risk trajectories in men aged <65 years and lower severe-risk trajectories in those aged ≥65 years.

**Conclusion:**

Sex- and age-specific behavioral trajectories were differentially associated with healthcare utilization and expenditure, underscoring the importance of multidimensional behavioral patterns in developing preventive strategies to reduce healthcare burdens.

## Introduction

Non-communicable diseases have become a dominant driver of healthcare demand globally, necessitating prevention-oriented health system reforms [1]. In Korea, Non-communicable diseases account for approximately 71 trillion KRW annually, nearly 85% of total healthcare costs, and healthcare expenditure has been growing at the fastest rate among Organization for Economic Co-operation and Development (OECD) countries, placing significant pressure on the long-term financial sustainability of the health system [2].

Korea is, however, uniquely positioned to address this challenge: it has long operated a universal single-payer system administered by the National Health Insurance Service (NHIS), which achieved near-universal population coverage (approximately 97%) as early as 1989 [3]. The NHI broadly comprises two enrollment types, workplace-based insurance for salaried employees and region-based insurance for the self-employed, while the remaining 3% of the population is covered by the tax-funded Medical Aid Program for those unable to afford contributions, analogous to Medicaid in the United States [4]. A notable strength of this system is the availability of individual-level insurance claims data linked to enrollment and premium information; because premiums are assessed according to income and assets, NHIS premium deciles serve as a validated income proxy and are widely used in Korean epidemiological research [4].

Despite these structural strengths, the system is not immune to the financial pressures of preventable disease: out-of-pocket spending remains above the OECD average, and Non-communicable diseases-driven costs continue to escalate [2]. Understanding how long-term health behavior patterns shape healthcare utilization within this system is therefore essential for informing cost-containment strategies and sustaining the NHI’s financial viability.

Meanwhile, Health behaviors (HBs) function as key modifiable drivers of healthcare utilization [5]. Empirical evidence indicates that smoking, heavy alcohol use, obesity, and physical inactivity are each independently associated with substantially higher healthcare expenditures and utilization [6, 7]. However, single-behavior approaches fail to capture the clustering and interactive effects of co-occurring risk behaviors [8], and most existing studies rely on cross-sectional designs that cannot account for the cumulative effects of long-term behavioral patterns on healthcare demand [7]. Furthermore, existing evidence has not adequately differentiated whether behavioral risk trajectories primarily affect total expenditure, service frequency, or care intensity as reflected in hospitalization patterns. These gaps are particularly relevant in South Korea, where smoking prevalence, high-risk drinking, physical inactivity, and obesity remain below OECD benchmarks [2, 9], and the associated socioeconomic losses reached approximately 42 trillion KRW in 2019 [10].

By integrating group-based multi-trajectory modeling with national insurance claims data, this study aims to provide evidence informing prevention-oriented policy to reduce healthcare burden in Korea. Specifically, three research questions were addressed: (1) What distinct long-term trajectories of composite HB change characterize middle-aged and older Korean adults? (2) Do these trajectories differentially predict healthcare expenditure, outpatient utilization, and hospitalization? (3) Is the association between unhealthy lifestyle trajectories and healthcare utilization explained by service frequency, care intensity, or both?

## Methods

### Data source and study population

The NHIS-HEALS Cohort is an administrative claims-based cohort — not a survey dataset — comprising a 10% random sample (n = 514,866) of Korean adults aged 40–79 who underwent national health screening in 2002–2003 [3]. As an administrative database, NHIS-HEALS is not subject to the recall bias or measurement error inherent in self-reported survey data, thereby strengthening the validity of the exposure and outcome measures used in this study. The NHIS, which covers approximately 97% of Korea’s population, mandates biennial health screenings for all enrollees (annual for manual workers), with each screening wave assessing anthropometry, laboratory markers, and health behaviors. Data were collected across four biennial waves: 2002–2003, 2004–2005, 2006–2007, and 2008–2009. These four waves constituted the trajectory estimation period, during which longitudinal changes in smoking, alcohol consumption, physical activity (PA), and Body Mass Index (BMI) were modeled. Healthcare expenditure and utilization outcomes were subsequently measured over the follow-up period from 2009 to 2019. NHIS-HEALS provides longitudinal linkage across screening records, insurance eligibility, income, demographics, medical treatments, and mortality data including cause and date of death [3].

Of the 514,789 individuals enrolled in 2002–2003, participants were excluded in three sequential steps. First, 43,676 former smokers and 40,242 individuals with baseline diagnoses of tuberculosis, hepatitis, liver disease, hypertension, heart disease, stroke, diabetes, or cancer were excluded, as these conditions may independently influence both health behaviors and healthcare utilization. Second, 258,970 individuals were excluded due to missing values in any of the four health behavior variables across the trajectory estimation waves or death during this period. Third, a further 4,558 individuals were excluded due to missing covariate data at the 2008–2009 wave (residential area or Charlson Comorbidity Index (CCI)). The final analytic sample comprised 95,226 males and 72,117 females, and a flow diagram of the sample selection process is presented in Supplementary Table S1.

Attrition across waves was primarily attributable to non-participation in scheduled screenings and mortality, both of which may be non-random. To assess the potential impact of selective attrition on representativeness, we compared the distribution of key covariates between included and excluded participants, with results presented in Supplementary Table S2. In the male group, statistically significant differences were observed in age and income level between included and excluded participants. In the female group, differences were observed in age, income level, disability status, and CCI. These findings suggest that the analytical sample may be somewhat healthier and more socioeconomically advantaged than the broader eligible population, and this selective attrition should be considered when interpreting the generalizability of findings.

### Independent variables

#### Health Behavior Trajectory (HBT)

To identify HBT, two analytic steps were applied: (1) categorization of HB variables and (2) Group-Based Multi-Trajectory Modeling (GBMTM). Four HB factors were included—tobacco use, alcohol consumption, PA, and BMI. Tobacco use was classified into five levels [11]: 0, 1–9, 10–19, 20–39, and ≥40 cigarettes/day. Alcohol intake, calculated in grams/day from questionnaire data, was categorized as 0, 1–9.9, 10–19.9, 20–29.9, and ≥30 g/day [12]. PA was assessed by weekly frequency of moderate-to-vigorous exercise [13]: none, 1–2, 3–4, 5–6 times, or almost daily. BMI was grouped by Korean Obesity Society guidelines [14]: underweight (<18.5), normal (18.5–22.9), overweight (23.0–24.9), obese (25.0–29.9), and severely obese (≥30.0 kg/m^2^).

Using these categories, GBMTM estimated trajectories over 2002/2003–2008/2009. Model fit was assessed by Akaike Information Criterion (AIC) [15], Bayesian Information Criterion (BIC) [15], entropy [16], and Average Posterior Probability (APP) [17], with a six-group solution selected as optimal for both males and females (Supplementary Table S3).

### Dependent variables

#### Healthcare expenditure and utilization

In this study, dependent variables were healthcare expenditures and utilization derived from NHIS-HEALS claims data [18]. Total Medical Expenditure (TME) was divided into inpatient TME (InTME) and outpatient TME (OutTME), while utilization included Length of Stay (LOS) and the number of outpatient visits.

Pharmacy costs (prescription medications dispensed at community pharmacies) were excluded from the expenditure calculations for the following reasons. First, the primary objective of this study was to examine the association between HBT and healthcare service utilization and expenditure, rather than medication adherence or pharmaceutical consumption patterns. Including pharmacy costs would have shifted the analytical focus toward downstream pharmacological management of disease, which was beyond the scope of the present study.

Second, and most critically, within the NHIS claims data utilized in this study, pharmaceutical expenditure is not disaggregated by care setting [11]. The available data capture only total prescription and dispensing costs as a combined figure, without any variable that distinguishes whether the costs were incurred in the context of outpatient or inpatient care. Since the present study separately examines inpatient and outpatient expenditure as distinct outcome variables, the inability to attribute pharmacy costs to either care setting precluded their inclusion in a methodologically sound manner. Any operational classification of pharmaceutical expenditure into outpatient- and inpatient-linked components would necessarily involve substantial uncertainty and arbitrary assumptions, which could introduce greater bias than the exclusion itself and compromise the analytical validity of the expenditure outcomes.

Third, in the Korean healthcare context, pharmacy claims represent a distinct dimension of healthcare behavior that is partially independent of physician-initiated care decisions, as patients may obtain over-the-counter medications or choose not to fill prescribed medications [19]. This further complicates the interpretation of pharmacy costs as a direct measure of healthcare service demand driven by disease occurrence and severity.

Fourth, exclusion of pharmacy costs allows for clearer interpretation of the relationship between lifestyle patterns and direct healthcare service demand — specifically, physician consultations, diagnostic procedures, and hospital admissions — which are more proximally influenced by disease occurrence and severity, and thus more directly relevant to the research questions of the present study.

Nevertheless, we acknowledge that this exclusion may lead to a systematic underestimation of the total financial burden attributable to unhealthy behavioral trajectories, particularly with respect to chronic disease management, where prescription medications constitute a substantial proportion of total healthcare costs — accounting for approximately 23.9% of total healthcare expenditure in Korea over the 2011–2020 period [11]. Future research should address this limitation by incorporating pharmaceutical expenditure once clearly defined and validated methods for pharmaceutical cost attribution by care setting are established in the literature.

### Control variables

Potential confounders included age, region, medical insurance, income, disability, and CCI, all of which may affect healthcare expenditure and utilization.

Age was categorized as 40–54, 55–64, 65–74, 75–84, and ≥85 years. Regions were classified into three groups: the Seoul Capital Area (Seoul, Incheon, Gyeonggi Province), metropolitan cities (Busan, Daegu, Daejeon, Gwangju, Ulsan), and other provinces (Gangwon, Chungbuk, Chungnam, Jeonbuk, Jeonnam, Gyeongbuk, Gyeongnam, Jeju). Medical insurance was grouped into National Health Insurance (region-based), National Health Insurance (workplace-based), and Medical Aid.

Income level was defined using NHIS household insurance premium deciles: deciles 0–3 (low), 4–7 (middle), and 8–10 (high). Disability status was assessed by medical specialists according to the Enforcement Rules of the Welfare of Persons with Disabilities Act. Before 2016, grades 3–6 were “Mild” and grades 1–2 “Severe”; after revision in 2016, grades 4–6 were classified as “Mild” and grades 1–3 as “Severe”.

The CCI, widely used in longitudinal studies, was calculated from ICD-10 codes for 19 comorbidities (myocardial, vascular, pulmonary, endocrine, renal, gastrointestinal, cancer/immune, and neurological), each weighted 1–6 points. Participants were divided into four groups: 0, 1, 2, and ≥3 points.

### Analytical approach and statistics

This study aimed to identify sex-specific HBTs and their associations with healthcare expenditure and utilization; the analytical framework is shown in Supplementary Table S4. GBMTM was chosen as the primary approach because it captures between-group heterogeneity by identifying qualitatively distinct subgroups with characteristic multi-behavioral trajectories, rather than describing average within-population change [17]. This makes it better suited than latent growth curve modeling—which models individual variation around a single population mean—for identifying discrete high-risk subgroups for targeted prevention [20].

For InTME and LOS, which exhibited substantial zero-inflation (approximately 80% and 84% zero observations in males, and 80% and 77% in females, respectively), we applied a two-part modeling framework [21]. The first part employed logistic GEE with a logit link function to model the probability of any inpatient utilization (versus zero), and the second part used GEE with a log link function and gamma distribution to model the conditional magnitude of expenditure or length of stay among those with non-zero values. This approach appropriately handles the semi-continuous nature of the data and avoids bias introduced by the retransformation problem inherent in log-transformed ordinary least squares regression [21].

Marginal effects representing the combined impact of both parts were calculated and presented (Equations 1-4). Specifically, the predicted probability of any inpatient utilization for each trajectory group (*P*_group_) was derived from Part 1 as:

$$P_{group}=\left(P_{ref}\times \text{OR}\right)\;/\;\left(1-P_{ref}+P_{ref}\times \text{OR}\right)$$ (1)

and the conditional expected expenditure or length of stay (*E*[Y |Y > 0, group]) was derived from Part 2 as:

$$E\left[Y\;|\;Y\;>\;0,\text{group}\right]=E\left[Y\;|\;Y\;>\;0,\text{ref}\right]\times \exp\left(\beta \right)$$ (2)

where *P*_*ref*_ and *E*[Y |Y > 0, ref] denote the observed hospitalization rate and mean inpatient expenditure (or length of stay) of the stable healthy group, respectively. The marginal effect for each group was then computed as:

$$ME=P\_\left(group\right)\times E\left[Y\;|\;Y\;>\;0,\text{group}\right]$$ (3)

$$\Delta ME=ME_{group}-ME_{ref}$$ (4)

The difference from the reference group (ΔME) was reported as the primary marginal effect estimate. Part 1 and Part 2 results are presented separately alongside marginal effects in Supplementary Table S5.

For OutTME and the number of outpatient visits, the proportion of zero observations was exceptionally low (less than 0.3%), rendering a two-part model unnecessary. As these outcomes did not require decomposition into utilization probability and conditional magnitude, only second-part results are presented: a single gamma GEE with log link was applied to OutTME, and a negative binomial GEE with log link was applied to the number of outpatient visits, given its discrete nature and overdispersion (variance exceeding the mean) [22].

The selection of correlation structure for GEE models was determined through systematic comparison of candidate structures based on quasi-likelihood under the independence model criterion. We evaluated four correlation structures for each outcome. The autoregressive order 1 (AR-1) structure was selected as it consistently yielded the lowest model fit criteria values across models, indicating superior model fit. The AR-1 structure is theoretically appropriate for this dataset given that healthcare expenditure and utilization in a given year are more strongly correlated with the immediately preceding year than with more distant time points [23]. All GEE models were adjusted for potential confounders including age, residential region, type of medical insurance, income level, disability status, and CCI.

Data management and statistical analyses were performed using SAS Enterprise Guide 8.3 (SAS Institute Inc., Cary, NC, USA) and R Studio 4.3.0 (R Studio Inc., Boston, MA, USA). Statistical significance was set at p < 0.05.

## Results

### Trajectory class of HB by sex over time

Figure 1 shows the six HBT identified among 95,226 males during 2002–2009, with 95% CI for each trajectory. The stable healthy group (30.3%) maintained persistent non-smoking, low alcohol intake, increasing PA, and normal BMI. The decreasing-risk group (28.0%) showed progressive improvement across all behaviors, including smoking cessation, reduced alcohol intake, increased PA, and declining BMI. The lower and higher moderate-risk groups (20.8% and 19.0%) shared sustained smoking, moderate drinking, and low PA, but differed in BMI, with the former maintaining overweight BMI and the latter normal BMI. The lower severe-risk group (0.9%) was characterized by light smoking, low alcohol intake, minimal PA, and persistent underweight, whereas the higher severe-risk group (1.1%) showed a similar smoking and activity profile but persistent severe obesity. The largely non-overlapping CI across major trajectory clusters support the statistical distinguishability of the six-group solution (Supplementary Table S6).

**FIGURE 1:**
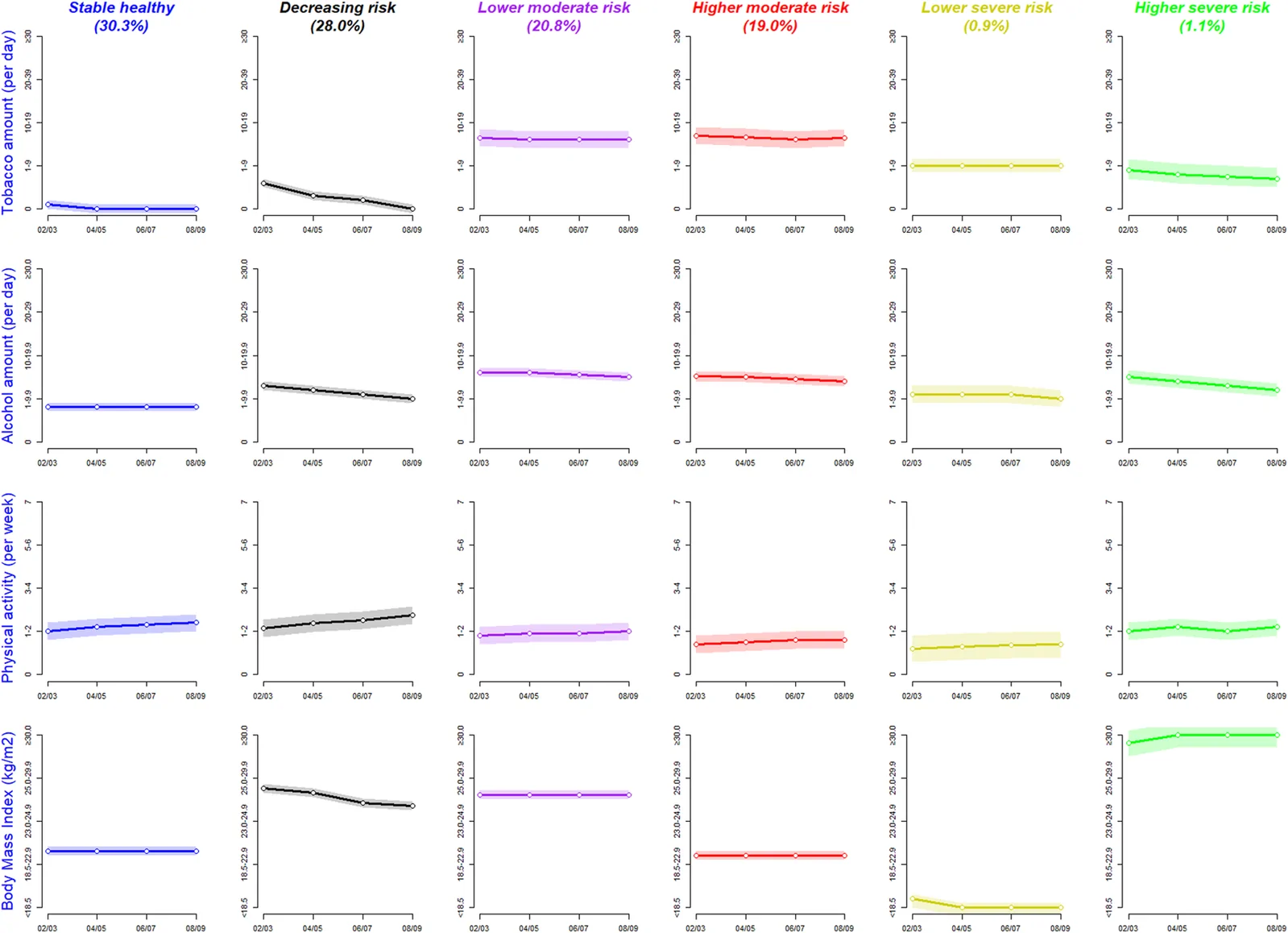
Trajectories of health behaviors among males, NHIS-HEALS Cohort (South Korea, 2002–2019).

Among 72,117 females, six HBT were also identified (Figure 2), with 95% CI presented for each trajectory. The stable healthy group (34.7%) maintained non-smoking, non-drinking, increasing PA, and normal BMI throughout follow-up. The decreasing-risk group (6.8%) showed reduced alcohol intake, increased PA, and declining BMI while remaining non-smoking. The lower and higher moderate-risk groups (23.8% and 23.5%) were both characterized by non-smoking, non-drinking, and increasing PA, but differed by persistently overweight versus obese BMI. The lower severe-risk group (10.2%) showed light smoking, low alcohol intake, low PA, and normal BMI, whereas the higher severe-risk group (0.9%) showed similar behavioral patterns but persistently higher BMI. Overall, the CI indicated sufficient separation across the female trajectory groups, particularly for BMI-defined clusters, supporting the six-group solution (Supplementary Table S7).

**FIGURE 2:**
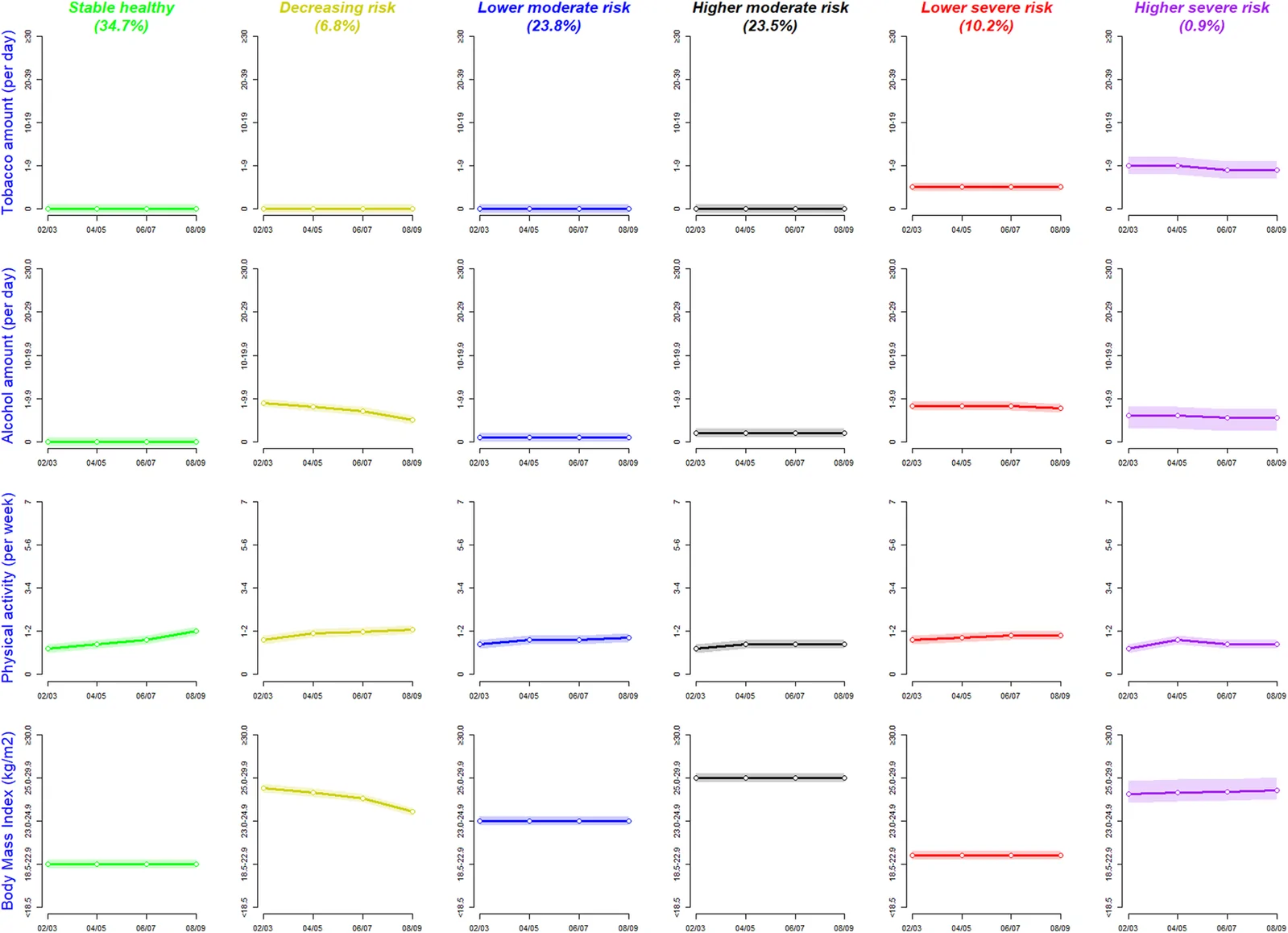
Trajectories of health behaviors among females, NHIS-HEALS Cohort (South Korea, 2002–2019).

### Sample characteristics by sex

Table 1 summarizes healthcare expenditure and utilization in the male cohort (n = 95,226). The mean OutTME and InTME values were 413,895 KRW (SD ±943,053) and 343,943 KRW (SD ±1,998,245), respectively. The average no. of outpatient visits was 18.35 (SD ±21.21), and mean LOS was 1.74 days (SD ±1.74). All variables differed significantly across the HBT (p < 0.0001).

**TABLE 1:** General characteristics of health behavior trajectories and healthcare variables among males at the baseline (2008–2009), NHIS‐HEALS Cohort (South Korea, 2002–2019).

Variables	Total		OutTME		P-value	InTME		P-value	No. of outpatient visit		P-value	LOS		P-value
	N	%	Mean	SD	​	Mean	SD	​	Mean	SD	​	Mean	SD	​
Total	95,226	(100.0)	413,895	(943,053)	​	343,943	(1,998,245)	​	18.35	(21.21)	​	1.74	(1.74)	​
Health behavior trajectory	​	​	​	​	<0.0001	​	​	<0.0001	​	​	<0.0001	​	​	<0.0001
Stable healthy	28,819	(30.3)	437,768	828,233	​	381,699	2,139,697	​	19.94	(23.28)	​	1.97	(11.85)	​
Decreasing risk	26,658	(28.0)	432,735	1,245,302	​	355,502	1,923,937	​	18.82	(21.47)	​	1.75	(10.40)	​
Lower moderate risk	19,771	(20.8)	370,856	752,326	​	278,073	1,990,217	​	15.98	(18.30)	​	1.44	(8.77)	​
Higher moderate risk	18,076	(19.0)	390,080	765,556	​	326,765	1,830,363	​	17.50	(19.83)	​	1.61	(9.16)	​
Lower severe risk	871	(0.9)	557,856	1,241,976	​	548,844	2,275,640	​	23.77	(25.93)	​	3.13	(14.88)	​
Higher severe risk	1,031	(1.1)	369,998	531,442	​	367,143	2,419,883	​	17.16	(18.82)	​	2.01	(13.80)	​
Age	​	​	​	​	<0.0001	​	​	<0.0001	​	​	<0.0001	​	​	<0.0001
40–54	47,734	(50.1)	301,181	678,932	​	206,863	1,502,197	​	12.84	(14.35)	​	1.11	(7.31)	​
55–64	31,481	(33.1)	429,859	893,883	​	346,371	2,182,044	​	18.39	(18.15)	​	1.69	(10.21)	​
65–74	12,116	(12.7)	669,942	1,573,352	​	671,283	2,607,183	​	32.24	(31.28)	​	3.30	(15.62)	​
75–84	3,810	(4.0)	796,278	1,065,614	​	903,696	2,937,514	​	38.80	(34.79)	​	4.73	(18.69)	​
≥85	85	(0.1)	968,270	1,233,064	​	637,079	1,776,905	​	44.26	(36.15)	​	4.44	(17.95)	​
Region	​	​	​	​	0.139	​	​	0.882	​	​	0.012	​	​	0.827
Capital area	39,819	(41.8)	417,525	1,041,987	​	350,023	2,165,094	​	18.62	(21.53)	​	1.79	(10.76)	​
Metropolitan area	22,238	(23.4)	394,172	790,859	​	324,195	1,803,970	​	17.45	(20.04)	​	1.67	(10.84)	​
Other areas	33,169	(34.8)	422,751	911,019	​	349,873	1,911,370	​	18.64	(21.56)	​	1.73	(9.74)	​
Medical insurance	​	​	​	​	0.033	​	​	0.545	​	​	<0.0001	​	​	0.803
NHI (region)	14,418	(15.1)	498,462	1,043,727	​	443,446	2,248,415	​	22.51	(25.10)	​	2.15	(10.98)	​
NHI (workplace)	80,741	(84.8)	398,512	923,097	​	325,851	1,949,613	​	17.60	(20.33)	​	1.67	(10.34)	​
Medical aid	67	(0.1)	511,357	589,800	​	449,028	1,164,230	​	24.05	(17.91)	​	2.02	(5.01)	​
Income level	​	​	​	​	0.414	​	​	0.619	​	​	0.415	​	​	0.855
Low	13,222	(13.9)	481,673	1,012,298	​	433,526	2,610,346	​	21.27	(23.17)	​	2.13	(12.32)	​
Mid	26,196	(27.5)	423,135	848,144	​	356,073	1,981,325	​	19.14	(21.79)	​	1.80	(10.58)	​
High	55,808	(58.6)	393,231	967,252	​	316,670	1,828,636	​	17.28	(20.33)	​	1.62	(9.86)	​
Disability	​	​	​	​	0.785	​	​	0.953	​	​	0.042	​	​	0.527
No	87,387	(91.8)	408,620	962,133	​	336,436	1,995,018	​	18.00	(20.84)	​	1.69	(10.27)	​
Mild	6,041	(6.3)	468,209	698,501	​	416,387	1,987,216	​	22.03	(24.78)	​	2.26	(12.09)	​
Severe	1,798	(1.9)	484,516	691,450	​	460,682	2,174,422	​	23.04	(23.81)	​	2.33	(12.08)	​
CCI	​	​	​	​	0.743	​	​	0.881	​	​	<0.0001	​	​	0.255
0	65,317	(68.6)	395,585	849,921	​	324,744	2,001,082	​	17.32	(19.88)	​	1.67	(10.25)	​
1	24,370	(25.6)	439,264	1,152,896	​	369,783	1,964,282	​	19.79	(22.85)	​	1.79	(10.20)	​
2	4,736	(5.0)	510,804	970,334	​	453,713	2,106,220	​	23.68	(26.29)	​	2.42	(13.48)	​
≥3	803	(0.8)	546,424	782,543	​	457,513	2,097,961	​	26.80	(28.79)	​	2.41	(11.31)	​

OutTME: Outpatient total medical expenditure/InTME: Inpatient total medical expenditure/LOS: Length of stay/NHI: National Health Insurance/CCI: charlson comorbidity index.

In the stable healthy group, OutTME was 437,768 KRW (SD ±943,053), InTME 381,699 KRW (SD ±2,139,697), outpatient visits 19.94 (SD ±23.28), and LOS 1.97 days (SD ±11.85). In contrast, the lower severe-risk group showed the highest values: OutTME 557,856 KRW (SD ±1,241,976), InTME 548,844 KRW (SD ±2,275,640), outpatient visits 23.77 (SD ±15.93), and LOS 3.13 days (SD ±14.88).

Table 2 summarizes the healthcare variables across the HBT in the female cohort (n = 72,117). The mean OutTME and InTME were 516,161 KRW (SD ±716,633) and 340,273 KRW (SD ±1,646,449), with 25.60 outpatient visits (SD ±24.74) and LOS of 2.03 days (SD ±10.43). All variables differed significantly across the HBT (p < 0.0001). OutTME and outpatient visits were highest in the higher moderate-risk group (558,548 KRW, SD ±720,051; 28.22 visits, SD ±26.26), whereas InTME and LOS peaked in the higher severe risk-group (462,237 KRW, SD ±2,017,844; 2.68 days, SD ±16.85).

**TABLE 2:** General characteristics of health behavior trajectories and healthcare variables among females at the baseline (2008–2009), NHIS‐HEALS Cohort (South Korea, 2002–2019).

Variables	Total		OutTME		P-value	InTME		P-value	No. of outpatient visit		P-value	LOS		P-value
	N	%	Mean	SD	​	Mean	SD	​	Mean	SD	​	Mean	SD	​
Total	72,117	(100.0)	516,161	(716,633)	​	340,273	(1,646,449)	​	25.60	(24.74)	​	2.03	(10.43)	​
Health behavior trajectory	​	​	​	​	<0.0001	​	​	0.000	​	​	<0.0001	​	​	0.039
Stable healthy	25,045	(34.7)	498,254	681,318	​	324,162	1,626,960	​	24.69	24.05	​	1.95	10.34	​
Decreasing risk	4,927	(6.8)	482,763	861,559	​	316,914	1,677,294	​	22.99	21.72	​	1.95	9.58	​
Lower moderate risk	17,198	(23.8)	532,625	723,138	​	352,702	1,608,696	​	26.39	25.42	​	2.13	10.51	​
Higher moderate risk	16,916	(23.5)	558,548	720,051	​	376,412	1,791,162	​	28.22	26.26	​	2.16	10.61	​
Lower severe risk	7,382	(10.2)	461,360	713,914	​	287,333	1,367,715	​	22.39	22.85	​	1.80	9.88	​
Higher severe risk	649	(0.9)	534,137	502,053	​	462,237	2,017,844	​	27.68	25.26	​	2.68	16.85	​
Age	​	​	​	​	<0.0001	​	​	<0.0001	​	​	<0.0001	​	​	<0.0001
40–54	31,309	(43.4)	387,485	646,241	​	197,681	1,106,446	​	18.12	17.51	​	1.34	7.37	​
55–64	25,974	(36.0)	518,583	660,380	​	321,572	1,613,130	​	25.38	21.80	​	1.93	9.52	​
65–74	11,149	(15.5)	769,502	875,925	​	616,520	2,323,487	​	40.57	32.36	​	3.13	13.37	​
75–84	3,633	(5.0)	802,388	823,133	​	820,193	2,664,592	​	43.97	36.00	​	5.25	21.44	​
≥85	52	(0.1)	692,406	544,522	​	715,144	1,882,543	​	45.27	44.19	​	4.35	14.11	​
Region	​	​	​	​	0.590	​	​	0.457	​	​	0.713	​	​	0.963
Capital area	29,987	(41.6)	513,838	729,507	​	332,398	1,566,807	​	25.35	24.32	​	1.99	10.12	​
Metropolitan area	15,872	(22.0)	499,643	633,807	​	313,394	1,481,763	​	24.74	23.50	​	1.97	10.48	​
Other areas	26,258	(36.4)	528,773	747,988	​	365,466	1,820,018	​	26.41	25.88	​	2.12	10.73	​
Medical insurance	​	​	​	​	0.249	​	​	0.354	​	​	0.138	​	​	0.259
NHI (region)	19,272	(26.7)	548,602	717,712	​	389,759	1,896,480	​	27.53	26.07	​	2.28	11.51	​
NHI (workplace)	52,727	(73.1)	504,178	716,362	​	321,765	1,537,385	​	24.88	24.18	​	1.94	9.91	​
Medical aid	118	(0.2)	551,999	446,564	​	497,859	3,501,094	​	32.87	27.16	​	3.92	29.18	​
Income level	​	​	​	​	0.832	​	​	0.827	​	​	0.121	​	​	0.470
Low	16,979	(23.5)	514,880	747,708	​	348,096	1,660,887	​	25.42	24.23	​	2.15	11.73	​
Mid	24,640	(34.2)	505,789	699,413	​	324,391	1,637,771	​	24.94	24.24	​	1.93	10.07	​
High	30,498	(42.3)	525,235	712,582	​	348,724	1,645,321	​	26.24	25.39	​	2.05	9.92	​
Disability	​	​	​	​	0.887	​	​	0.921	​	​	0.660	​	​	0.681
No	67,669	(93.8)	510,328	717,110	​	332,844	1,634,477	​	25.23	24.45	​	1.99	10.28	​
Mild	3,631	(5.0)	609,661	730,095	​	459,122	1,878,074	​	31.28	27.81	​	2.66	12.91	​
Severe	817	(1.1)	581,131	570,602	​	424,019	1,499,777	​	30.94	29.74	​	2.73	10.15	​
CCI	​	​	​	​	0.434	​	​	0.003	​	​	0.022	​	​	0.625
0	44,009	(61.0)	496,956	710,373	​	310,405	1,451,012	​	24.40	23.62	​	1.90	9.60	​
1	21,672	(30.1)	532,913	713,424	​	363,111	1,776,820	​	26.77	25.88	​	2.16	11.26	​
2	5,472	(7.6)	583,347	777,472	​	438,111	2,179,470	​	29.37	27.17	​	2.47	12.57	​
≥3	964	(1.3)	631,288	667,453	​	629,971	2,908,627	​	32.66	28.71	​	2.90	13.14	​

OutTME: Outpatient total medical expenditure/InTME: Inpatient total medical expenditure/LOS: Length of stay/NHI: National Health Insurance/CCI: charlson comorbidity index.

### Adjusted association between HBT and healthcare expenditure/utilization

Table 3 presents the associations between HBT and healthcare variables in both cohorts. In males, the higher severe-risk trajectory showed significantly higher expenditures than the stable healthy group: OutTME +12.5% (B = 0.118, 95% CI: 0.07–0.17, p < 0.0001) and InTME +60.6% (B = 0.474, 95% CI: 0.26–0.68, p < 0.0001). It was also associated with higher utilization: outpatient visits +4.0% (B = 0.039, 95% CI: 0.01–0.07, p = 0.005) and LOS 22.0% (B = 0.199, 95% CI: 0.08–0.32, p = 0.001). The higher moderate-risk group showed fewer visits (−3.7%, B = −0.038, 95% CI: −0.05 to −0.03, p < 0.0001) but longer LOS (+24.4%, B = 0.218, 95% CI: 0.18–0.26, p < 0.0001). The decreasing-risk group had 4.8% fewer visits (B = −0.049, 95% CI: −0.08 to −0.02, p = 0.003).

**TABLE 3:** Adjusted effect of health behavior trajectories on inpatient healthcare variables by sex, NHIS‐HEALS Cohort (South Korea, 2002–2019).

Variables	OutTME					InTME					No. of outpatient visit					LOS				
	*B*	95% CI			P-value	*B*	95% CI			P-value	*B*	95% CI			P-value	*B*	95% CI			P-value
Health behavior trajectory	Male																			
Stable healthy	Ref	​	​	​	​	Ref	​	​	​	​	Ref	​	​	​	​	Ref	​	​	​	​
Decreasing risk	−0.068	(-0.14	-	0.00)	0.065	0.080	(0.04	-	0.12)	0.000	−0.049	(-0.08	-	−0.02)	0.003	−0.003	(-0.04	-	0.03)	0.850
Lower moderate risk	0.029	(0.01	-	0.05)	0.000	0.266	(0.22	-	0.31)	<0.0001	−0.007	(-0.01	-	0.00)	0.115	0.114	(0.08	-	0.15)	<0.0001
Higher moderate risk	−0.013	(-0.03	-	0.00)	0.122	0.293	(0.25	-	0.34)	<0.0001	−0.038	(-0.05	-	−0.03)	<0.0001	0.218	(0.18	-	0.26)	<0.0001
Lower severe risk	0.058	(0.04	-	0.07)	<0.0001	0.299	(0.14	-	0.45)	0.000	0.026	(0.02	-	0.03)	<0.0001	0.241	(0.12	-	0.37)	0.000
Higher severe risk	0.118	(0.07	-	0.17)	<0.0001	0.474	(0.26	-	0.68)	<0.0001	0.039	(0.01	-	0.07)	0.005	0.199	(0.08	-	0.32)	0.001
Health behavior trajectory	Female																			
Stable healthy	Ref	​	​	​	​	Ref	​	​	​	​	Ref	​	​	​	​	Ref	​	​	​	​
Decreasing risk	0.074	(0.05	-	0.10)	<0.0001	0.245	(0.19	-	0.30)	<0.0001	0.039	(0.03	-	0.05)	<0.0001	0.126	(0.08	-	0.17)	<0.0001
Lower moderate risk	0.054	(0.04	-	0.07)	<0.0001	0.113	(0.07	-	0.15)	<0.0001	0.042	(0.03	-	0.05)	<0.0001	0.054	(0.02	-	0.09)	0.002
Higher moderate risk	0.088	(0.07	-	0.10)	<0.0001	0.240	(0.20	-	0.28)	<0.0001	0.064	(0.06	-	0.07)	<0.0001	0.110	(0.08	-	0.14)	<0.0001
Lower severe risk	−0.010	(-0.03	-	0.01)	0.297	0.091	(0.03	-	0.15)	0.003	−0.014	(-0.02	-	0.00)	0.005	0.055	(0.01	-	0.10)	0.015
Higher severe risk	0.138	(0.08	-	0.20)	<0.0001	0.559	(0.43	-	0.69)	<0.0001	0.058	(0.03	-	0.09)	0.000	0.374	(0.24	-	0.51)	<0.0001

* All covariates were controlled/OutTME: Outpatient total medical expenditure/InTME: Inpatient total medical expenditure/LOS: length of stay.

In females, the higher severe-risk trajectory was linked to higher expenditures: OutTME +14.8% (B = 0.138, 95% CI: 0.08–0.20, p < 0.0001) and InTME +74.7% (B = 0.558, 95% CI: 0.43–0.69, p < 0.0001). Utilization also rose: visits +6.0% (B = 0.058, 95% CI: 0.03–0.09, p = 0.000) and LOS +45.4% (B = 0.374, 95% CI: 0.24–0.51, p < 0.0001). The decreasing-risk group also showed higher expenditures and utilization than the reference group.

### Stratified analysis of HBT and healthcare expenditure/utilization

Table 4 presents the age-stratified associations in males. Among those <65 years, both the lower and higher severe-risk trajectories were associated with higher InTME (B = 0.386, 95% CI: 0.16–0.61, p = 0.001; B = 0.543, 95% CI: 0.28–0.81, p < 0.0001) and longer LOS (B = 0.138, 95% CI: 0.10–0.44, p = 0.002; B = 0.250, 95% CI: 0.11–0.39, p = 0.000) compared to the stable healthy group. Among those ≥65 years, both the lower and higher moderate-risk trajectories were similarly associated with higher InTME (B = 0.258, 95% CI: 0.19–0.32, p < 0.0001; B = 0.318, 95% CI: 0.26–0.38, p < 0.0001) and longer LOS (B = 0.183, 95% CI: 0.11–0.25, p < 0.0001; B = 0.273, 95% CI: 0.20–0.34, p < 0.0001).

**TABLE 4:** Stratified analysis of the association between health behavior trajectories and healthcare variables by sex and age, NHIS‐HEALS Cohort (South Korea, 2002–2019).

Variables	OutTME				InTME				No. of outpatient visit				LOS			
	*B*	95% CI		P-value	*B*	95% CI		P-value	*B*	95% CI		P-value	*B*	95% CI		P-value
Health behavior trajectory	Male aged ≤64y															
Stable healthy	Ref	​	​	​	Ref	​	​	​	Ref	​	​	​	Ref	​	​	​
Decreasing risk	−0.049	(-0.15	0.05)	0.342	0.101	(0.04	0.16)	0.001	−0.048	(-0.09	0.00)	<0.0001	0.013	(-0.03	0.05)	0.499
Lower moderate risk	0.04	(0.02	0.06)	<0.0001	0.277	(0.22	0.33)	<0.0001	0.014	(0.00	0.02)	0.029	0.104	(0.07	0.14)	<0.0001
Higher moderate risk	−0.011	(-0.03	0.01)	0.284	0.285	(0.23	0.34)	<0.0001	−0.031	(-0.04	−0.02)	<0.0001	0.199	(0.15	0.24)	<0.0001
Lower severe risk	0.071	(0.05	0.09)	<0.0001	0.386	(0.16	0.61)	0.001	0.042	(0.03	0.05)	<0.0001	0.273	(0.10	0.44)	0.002
Higher severe risk	0.136	(0.08	0.20)	<0.0001	0.543	(0.28	0.81)	<0.0001	0.079	(0.05	0.11)	<0.0001	0.250	(0.11	0.39)	0.000
Health behavior trajectory	Male aged ≥65y															
Stable healthy	Ref	​	​	​	Ref	​	​	​	Ref	​	​	​	Ref	​	​	​
Decreasing risk	−0.123	(-0.19	−0.06)	0.000	0.049	(0.00	0.10)	0.065	−0.071	(-0.11	−0.03)	0.001	−0.026	(-0.08	0.03)	0.335
Lower moderate risk	0.051	(0.02	0.08)	0.001	0.258	(0.19	0.32)	<0.0001	−0.002	(-0.02	0.02)	0.86	0.183	(0.11	0.25)	<0.0001
Higher moderate risk	−0.003	(-0.03	0.03)	0.872	0.318	(0.26	0.38)	<0.0001	−0.039	(-0.05	−0.02)	<0.0001	0.273	(0.20	0.34)	<0.0001
Lower severe risk	0.054	(0.03	0.08)	0.000	0.142	(-0.01	0.30)	0.074	0.024	(0.01	0.04)	0.000	0.181	(0.00	0.37)	0.053
Higher severe risk	0.108	(0.02	0.19)	0.011	0.246	(0.11	0.46)	0.002	0.003	(-0.05	0.06)	0.919	0.163	(-0.05	0.38)	0.136
Health behavior trajectory	Female aged ≤64y															
Stable healthy	Ref	​	​	​	Ref	​	​	​	Ref	​	​	​	Ref	​	​	​
Decreasing risk	0.077	(0.05	0.10)	<0.0001	0.28	(0.21	0.35)	<0.0001	0.05	(0.04	0.06)	<0.0001	0.19	(0.13	0.24)	<0.0001
Lower moderate risk	0.048	(0.03	0.06)	<0.0001	0.108	(0.05	0.16)	0.000	0.043	(0.03	0.05)	<0.0001	0.049	(0.01	0.09)	0.013
Higher moderate risk	0.099	(0.08	0.12)	<0.0001	0.26	(0.21	0.31)	<0.0001	0.068	(0.03	0.11)	0.001	0.115	(0.08	0.15)	<0.0001
Lower severe risk	−0.016	(-0.04	0.01)	0.150	0.083	(0.01	0.16)	0.024	−0.006	(-0.02	0.00)	0.254	0.053	(0.00	0.10)	0.036
Higher severe risk	0.134	(0.06	0.21)	0.001	0.624	(0.42	0.82)	<0.0001	0.083	(0.07	0.09)	<0.0001	0.439	(0.25	0.62)	<0.0001
Health behavior trajectory	Female aged ≥65y															
Stable healthy	Ref	​	​	​	Ref	​	​	​	Ref	​	​	​	Ref	​	​	​
Decreasing risk	0.081	(0.04	0.12)	<0.0001	0.16	(0.08	0.24)	<0.0001	0.044	(0.00	0.09)	0.046	−0.055	(-0.14	0.03)	0.207
Lower moderate risk	0.066	(0.05	0.09)	<0.0001	0.119	(0.07	0.17)	<0.0001	0.045	(0.03	0.06)	<0.0001	0.041	(-0.01	0.10)	0.150
Higher moderate risk	0.08	(0.06	0.10)	<0.0001	0.221	(0.17	0.27)	<0.0001	0.048	(0.04	0.06)	<0.0001	0.086	(0.03	0.14)	0.003
Lower severe risk	0.019	(-0.02	0.06)	0.388	0.142	(0.05	0.23)	0.002	−0.006	(-0.03	0.02)	0.565	0.106	(0.02	0.19)	0.019
Higher severe risk	0.15	(0.07	0.23)	0.000	0.477	(0.33	0.63)	<0.0001	0.061	(0.04	0.08)	<0.0001	0.301	(0.13	0.47)	0.001

* All covariates were controlled/OutTME: Outpatient total medical expenditure/InTME: Inpatient total medical expenditure/LOS: length of stay.

Table 4 also presents the results in females. Among those <65 years, the higher severe-risk trajectory was associated with higher InTME (B = 0.624, 95% CI: 0.42–0.82, p < 0.0001) and longer LOS (B = 0.439, 95% CI: 0.25–0.62, p < 0.0001) compared to the stable healthy group. Among those ≥65 years, the higher severe-risk trajectory was similarly associated with higher InTME (B = 0.477, 95% CI: 0.33–0.63, p < 0.0001) and longer LOS (B = 0.301, 95% CI: 0.13–0.47, p = 0.001).

In addition, considering that the BMI trajectory may be more appropriately conceptualized as a physiological outcome rather than a volitional health behavior, we performed a sensitivity analysis in which BMI was removed from the HBT definition and additionally included as an adjustment variable. The results are presented in Supplementary Table S8.

## Discussion

This study used the 2002–2019 NHIS-HEALS data to identify sex-specific HBT and assess their links with healthcare expenditure and utilization. From 2002 to 2009, five maintenance activities and one improvement trajectory were observed in both sexes. In males, the decreasing-risk trajectory reduced outpatient visits and LOS, whereas the higher moderate-risk trajectory showed fewer visits but a longer LOS. In females, the higher severe-risk trajectory was associated with higher OutTME, InTME, and greater healthcare utilization, while the decreasing-risk trajectory was also associated with higher expenditures and utilization compared to the stable healthy group. Among males, severe- (<65 years) and moderate-risk trajectories (≥65 years) were strongly associated with higher expenditures and utilization.

Clear sex differences in the HBT were identified, with males showing more adverse patterns, consistent with prior evidence. In the Asian context, smoking and drinking are socially accepted and embedded in male interactions, whereas females maintain more stable HBs because of their household roles [24]. For middle-aged men, tobacco and alcohol serve as means of social bonding and stress relief [25]. In contrast, women, shaped by sociocultural norms are more likely to adopt risk-avoidance and health-promoting behaviors [26].

Higher moderate-risk males showed lower outpatient but higher inpatient indicators than stable-healthy males, despite normal BMI, younger age, lower disability, and more CCI = 0 cases—reflecting poor smoking, drinking, and PA profiles. This matches prior findings that normal-weight individuals visit outpatient care less but hospitalize more (the “lean paradox”), driven by smoking, risky drinking, and limited preventive care [27]. Supplementary Table S9 shows 3.0% lower outpatient expenditure per visit but 5.0% higher inpatient expenditure per day, indicating heavier hospitalization and cost burdens and the need for preventive interventions even in normal-BMI groups [28].

The male decreasing-risk trajectory showed fewer outpatient visits and shorter LOS, consistent with global evidence that long-term HB improvement reduces expenditures, hospitalizations, outpatient use, and LOS [24]. HB improvement lowers NCD risk by easing chronic inflammation, oxidative stress, and dyslipidemia, improving blood pressure, glucose, lipids, body composition, and endothelial function [29]. Per the “compression of morbidity” theory [30], this may delay disease onset, shorten morbidity duration, and reduce healthcare utilization.

Most women were nonsmokers/nondrinkers, but the higher severe-risk trajectory showed elevated smoking, drinking, and overweight rates, consistent with evidence linking adverse HBs to greater healthcare use in females (e.g., 18.6% higher TME in smokers, 31.8% in daily drinkers, 46.5% in severely obese, 7.4% in inactive individuals [7]), driven partly by smoking’s estrogen-lowering effects [31] and women’s greater physiological susceptibility to alcohol-related harm [32]. In contrast, the female decreasing-risk trajectory paradoxically showed higher expenditure and utilization than the stable healthy group—unlike males, and contrary to evidence that behavioral improvement lowers costs [31]. This group’s lower baseline CCI (Supplementary Table S10) rules out higher baseline morbidity as the cause. Nonetheless, the following mechanisms may help explain this finding.

First, the observed pattern may reflect a health-conscious behavioral response rather than illness-driven change. Women who modify their lifestyles may simultaneously adopt broader health-promoting behaviors, which could paradoxically elevate short-term healthcare utilization [33]. However, we were unable to directly examine whether the increase in outpatient visits among the decreasing-risk group was attributable to preventive or screening-related consultations rather than illness visits within the present study design, and this remains a direction for future investigation.

Second, the benefits of behavioral improvement may require a longer time horizon to manifest as reductions in healthcare expenditure. Accumulated organ damage from prior unhealthy behaviors, including subclinical cardiovascular disease, hepatic injury, or musculoskeletal deterioration, may continue to generate healthcare costs even after behavioral modification [7]. In this context, the 11-year follow-up period of the present study may be insufficient to capture the full economic benefits of behavioral improvement, particularly given biological sex differences in the trajectory and pace of disease progression [31, 32].

Third, biological sex differences in the health consequences of behavioral change may contribute to this divergence. Estrogen-mediated protective effects in younger women may attenuate the observable health benefits of behavioral improvement during the follow-up period, whereas the withdrawal of such protection in perimenopausal and postmenopausal women may amplify residual effects of prior unhealthy behaviors on healthcare utilization [31, 32].

In males, age-stratified analysis showed severe-risk trajectories strongly linked to expenditures and utilization in those <65 years, while moderate-risk trajectories were more influential in those ≥65 years. The severe-risk groups had less smoking and drinking but extreme BMI profiles. Despite similar behaviors, younger (<65) overweight or underweight males had higher costs and utilization than older counterparts [34], likely due to early disease onset and treatment needs. In contrast, those ≥65 years were more often multimorbid, requiring ongoing care, explaining the observed patterns [34]. In contrast, moderate-risk trajectories showed higher smoking and drinking habits than the severe-risk groups. Given their cumulative effects, persistent smoking and heavy drinking in adults ≥65 years may cause lasting damage and greater healthcare use [35]. Studies also reported that late-life smoking and alcohol worsen comorbidities, increasing disease severity and hospitalization rates [35].

Globally, HB improvement policies have been recognized as cost-effective strategies for reducing healthcare resource utilization [36]. However, in South Korea, community-level health policies have focused primarily on interventions after disease onset to prevent disease progression, and preventive measures are largely directed towards specific disease groups. Consequently, the potential for excessive healthcare utilization in the general population remains a concern [24]. A study from Japan reported that community-based health promotion programs and prevention-oriented policies resulted in reduced healthcare expenditure [37]. In line with these findings, the effectiveness of preventive policies in South Korea could be enhanced by adopting similar approaches.

First, substantial sample exclusions (258,970 for missing data/death; 83,918 for baseline chronic conditions) likely produced a healthier-than-average sample; excluded participants differed significantly in age, income, and (in females) disability and CCI (Supplementary Table S2), warranting caution in generalization. Second, unmeasured confounders—diet, sleep, education, occupation, health literacy—may have influenced both behaviors and utilization. Third, PA was measured only by weekly frequency, without duration or intensity, limiting accurate classification; future studies should include minutes per session and MET values. Fourth, findings from Korea’s single-payer system may not generalize to other healthcare financing structures. Finally, since BMI was one of the trajectory indicators in the multi-trajectory model, the sensitivity analysis (BMI as covariate) does not confirm whether the same clusters would emerge if BMI were excluded from clustering; although paired subgroups (e.g., lower/higher moderate- or severe-risk) show subtle differences in smoking, alcohol, and PA, BMI trajectory remains their most distinguishing feature, so these subgroups’ distinctiveness and clinical relevance should be interpreted cautiously.

### Conclusions

This study identified sex-specific smoking, alcohol, PA, and BMI trajectories among middle-aged and older Koreans, with differential links to healthcare use. Six trajectories emerged in both sexes, showing clear sex- and age-related differences: decreasing-risk was linked to lower utilization in males but higher expenditure and utilization in females. Severe-risk trajectories showed stronger associations under age 65, moderate-risk trajectories at age ≥65. These findings indicated that healthcare demand is shaped by long-term, multidimensional behavioral patterns rather than by single risk factors. Preventive interventions should address not only high-risk groups but also individuals with a normal BMI who continue to smoke, drink, or remain inactive. Strengthening community-based prevention programs may help reduce the healthcare burden and support the sustainability of Korea’s healthcare system.

## Data Availability

The data used in this study are not directly available from the authors but can be accessed for analysis through a secure virtual room following a formal application process via the following link: https://nhiss.nhis.or.kr/en/z/a/001/lpza001m01en.do.
