# Supplementary material for: Joint trajectories of health behaviors and their associations with healthcare utilization and expenditure
Source: Int J Public Health. 2026 Sep 25;71:1609686. doi: 10.3389/ijph.2026.1609686 (PMC13669875; doi:10.3389/ijph.2026.1609686)
Supplement: Supplementary file 2 [file Table10.docx]

**Supplementary Table S10. Distribution of Charlson Comorbidity Index (CCI) by health behavior trajectory group in females (%) (South Korea, 2026)**

*CCI: Charlson Comorbidity Index. Bars represent the percentage of individuals in each CCI category within each health behavior trajectory group. Numbers above bars indicate group sample sizes.*
