# Supplementary material for: Joint trajectories of health behaviors and their associations with healthcare utilization and expenditure
Source: Int J Public Health. 2026 Sep 25;71:1609686. doi: 10.3389/ijph.2026.1609686 (PMC13669875; doi:10.3389/ijph.2026.1609686)
Supplement: Supplementary file 3 [file Table2.docx]

| **Supplementary Table S2. Comparison between the analytic and excluded samples by sex (South Korea, 2026)** | | | | | | | |
| --- | --- | --- | --- | --- | --- | --- | --- |
| **Male group** | | | | **Female group** | | | |
| **Variables** | **Non-excluded**  **sample**  **Proportion**  **(%)** | **Excluded**  **sample**  **Proportion**  **(%)** | **Diff.** | **Variables** | **Non-excluded**  **sample**  **Proportion**  **(%)** | **Excluded**  **sample**  **Proportion**  **(%)** | **Diff.** |
|  |  |  |  |  |  |  |  |
| **Age** |  |  |  | **Age** |  |  |  |
| 45-54 | **50.13** | **43.55** | ***** | 45-54 | **43.41** | **40.27** | ***** |
| 55-64 | **33.06** | **32.92** | ***** | 55-64 | **36.02** | **32.71** | ***** |
| 65-74 | **12.72** | **18.43** | ***** | 65-74 | **15.46** | **20.31** | ***** |
| ≥75 | **4.09** | **5.09** | ***** | ≥75 | **5.11** | **6.71** | ***** |
| **Region** |  |  |  | **Region** |  |  |  |
| Capital area | 41.82 | 42.29 |  | Capital area | 41.58 | 41.49 |  |
| Metropolitan area | 23.35 | 23.21 |  | Metropolitan area | 22.01 | 22.36 |  |
| Other areas | 34.83 | 34.50 |  | Other areas | 36.41 | 36.14 |  |
| **Medical insurance** |  |  |  | **Medical insurance** |  |  |  |
| NHI(Region) | 15.14 | 15.43 |  | NHI(Region) | 26.72 | 26.47 |  |
| NHI(Workplace) | 84.79 | 84.49 |  | NHI(Workplace) | 73.11 | 73.34 |  |
| Medical aid | 0.07 | 0.08 |  | Medical aid | 0.16 | 0.20 |  |
| **Income level** |  |  |  | **Income level** |  |  |  |
| Low | **13.88** | **19.00** | ***** | Low | **23.54** | **25.01** | ***** |
| Mid | **27.51** | **32.87** | ***** | Mid | **34.17** | **34.44** | ***** |
| High | **58.61** | **48.12** | ***** | High | **42.29** | **40.55** | ***** |
| **Disability** |  |  |  | **Disability** |  |  |  |
| No | 91.77 | 91.56 |  | No | **93.83** | **94.66** | ***** |
| Mild | 6.34 | 6.54 |  | Mild | **5.03** | **5.12** | ***** |
| Severe | 1.89 | 1.90 |  | Severe | **1.13** | **0.22** | ***** |
|  |  |  |  |  |  |  |  |
| **CCI** |  |  |  | **CCI** |  |  |  |
| 0 | 68.59 | 68.26 |  | 0 | **61.02** | **60.19** | ***** |
| 1 | 25.59 | 25.67 |  | 1 | **30.05** | **29.54** | ***** |
| 2 | 4.97 | 5.19 |  | 2 | **7.59** | **8.28** | ***** |
| ≥3 | 0.84 | 0.89 |  | ≥3 | **1.34** | **1.99** | * |
| **Total participants** | **95,226** | **121,884** |  | **Total participants** | **72,117** | **101,816** |  |

*** Participants who had already died or were unavailable for follow-up at baseline, as well as those for whom overall baseline characteristics could not be ascertained, were excluded from the excluded sample.**
