# Supplementary material for: Joint trajectories of health behaviors and their associations with healthcare utilization and expenditure
Source: Int J Public Health. 2026 Sep 25;71:1609686. doi: 10.3389/ijph.2026.1609686 (PMC13669875; doi:10.3389/ijph.2026.1609686)
Supplement: Supplementary file 4 [file Table3.docx]

**Supplementary Table S3. Model fit statistics for estimated patterns of trajectory (South Korea, 2026)**

| **Male** | | | | | | | |
| --- | --- | --- | --- | --- | --- | --- | --- |
| **Fit statistics*** | **2 classes** | **3 classes** | **4 classes** | **5 classes** | **6 classes** | **7 classes** | **8 classes** |
| **AIC** | -2,164,137 | -2,088,291 | -2,025,486 | -2,004,043 | **-1,987,074** | -1,990,400 | -1,998,212 |
| **BIC^2^** | -2,164,275 | -2,088,491 | -2,025,748 | -2,004,366 | **-1,987,388** | -1,990,691 | -1,998,439 |
| **BIC^3^** | -2,164,315 | -2,088,550 | -2,025,824 | -2,004,461 | **-1,987,480** | -1,990,776 | -1,998,526 |
| **Entropy** | 0.99 | 0.99 | 0.99 | 0.99 | **0.99** | 0.99 | 0.99 |
|  |  |  |  |  |  |  |  |
| **Average of Posterior Probability** | C1= 98.6% | C1= 95.6% | C1= 93.2% | C1= 95.2% | **C1= 98.2%** | C1= 94.8% | C1= 93.8% |
|  | C2= 98.4% | C2= 96.1% | C2= 96.8% | C2= 93.3% | **C2= 99.8%** | C2= 88.6% | C2= 95.1% |
|  |  | C3= 95.7% | C3= 98.1% | C3= 93.1% | **C3= 93.0%** | C3= 86.6% | C3= 94.0% |
|  |  |  | C4= 93.8% | C4= 97.7% | **C4= 96.6%** | C4= 96.8% | C4= 93.4% |
|  |  |  |  | C5= 93.1% | **C5= 99.8%** | C5= 90.3% | C5= 97.2% |
|  |  |  |  |  | **C6= 94.3%** | C6= 84.9% | C6= 95.0% |
|  |  |  |  |  |  | C7= 95.7% | C7= 91.3% |
|  |  |  |  |  |  |  | C8= 94.6% |
| **Female** | | | | | | | |
| **Fit statistics**** | **2 classes** | **3 classes** | **4 classes** | **5 classes** | **6 classes** | **7 classes** | **8 classes** |
| **AIC** | -1,280,043 | -1,193,721 | -1,168,064 | -1,151,961 | **-972,103** | -1,139,044 | -1,139,149 |
| **BIC^2^** | -1,280,172 | -1,193,910 | -1,168,313 | -1,152,288 | **-972,513** | -1,139,413 | -1,138,578 |
| **BIC^3^** | -1,280,211 | -1,193,967 | -1,168,388 | -1,152,386 | **-972,636** | -1,139,524 | -1,138,707 |
| **Entropy** | 0.99 | 0.99 | 0.99 | 0.99 | **0.99** | 0.99 | 0.99 |
|  |  |  |  |  |  |  |  |
| **Average of Posterior Probability** | C1= 96.2% | C1= 98.8% | C1= 95.4% | C1= 94.2% | **C1= 93.8%** | C1= 90.2% | C1= 89.4% |
|  | C2= 98.6% | C2= 88.9% | C2= 88.1% | C2= 95.4% | **C2= 94.0%** | C2= 93.8% | C2= 93.3% |
|  |  | C3= 95.6% | C3= 98.1% | C3= 96.7% | **C3= 95.8%** | C3= 96.7% | C3= 99.8% |
|  |  |  | C4= 90.0% | C4= 92.8% | **C4= 98.4%** | C4= 94.2% | C4= 92.4% |
|  |  |  |  | C5= 93.1% | **C5= 93.3%** | C5= 98.6% | C5= 94.1% |
|  |  |  |  |  | **C6= 96.2%** | C6= 94.4% | C6= 90.5% |
|  |  |  |  |  |  | C7= 92.9% | C7= 96.5% |
|  |  |  |  |  |  |  | C8= 94.1% |
| ***N=95,226 / **N=72,117 / AIC: Akaike Information Criterion / BIC: Bayesian Information Criterion** | | | | | | | |
