# Supplementary material for: Joint trajectories of health behaviors and their associations with healthcare utilization and expenditure
Source: Int J Public Health. 2026 Sep 25;71:1609686. doi: 10.3389/ijph.2026.1609686 (PMC13669875; doi:10.3389/ijph.2026.1609686)
Supplement: Supplementary file 6 [file Table5.docx]

| **Supplementary Table S5. Marginal effects of GEE estimations (South Korea, 2026)** | | | | | | | |
| --- | --- | --- | --- | --- | --- | --- | --- |
| **Variables** | **InTME** | | |  | **LOS** | | |
|  | **PART Ⅰ** | **PART Ⅱ** | **Marginal Effect** |  | **PART Ⅰ** | **PART Ⅱ** | **Marginal Effect** |
|  | **OR** | ***B*** | **Mean** |  | **OR** | ***B*** | **Mean** |
| **Health behavior trajectory** | **Male** | | | | | | |
| **Stable healthy** | 1.00 | *Ref* | *Ref* |  | 1.00 | *Ref* | *Ref* |
| **Decreasing risk** | 1.06*** | 0.08*** | 90,463*** |  | 1.05 | -0.003 | 0.138 |
| **Lower moderate risk** | 1.20*** | 0.27*** | 347,207*** |  | 1.19*** | 0.114*** | 0.948*** |
| **Higher moderate risk** | 1.19*** | 0.29*** | 359,073*** |  | 1.19*** | 0.218*** | 1.407*** |
| **Lower severe risk** | 1.17*** | 0.30*** | 351,121*** |  | 1.18*** | 0.241*** | 1.479*** |
| **Higher severe risk** | 1.24*** | 0.47*** | 612,074*** |  | 1.23*** | 0.199*** | 1.450*** |
| **Health behavior trajectory** | **Male aged ≤ 64y** | | | | | | |
| **Stable healthy** | 1.00 | *Ref* | *Ref* |  | 1.00 | *Ref* | *Ref* |
| **Decreasing risk** | 1.17*** | 0.10*** | 91,897*** |  | 1.09 | 0.013 | 0.140 |
| **Lower moderate risk** | 1.22*** | 0.28*** | 196,418*** |  | 1.22*** | 0.104*** | 0.473*** |
| **Higher moderate risk** | 1.20*** | 0.29*** | 193,512*** |  | 1.19*** | 0.199*** | 0.624*** |
| **Lower severe risk** | 1.10*** | 0.39*** | 209,345*** |  | 1.18*** | 0.273** | 0.769** |
| **Higher severe risk** | 1.29*** | 0.54*** | 390,902*** |  | 1.28*** | 0.250*** | 0.875** |
| **Health behavior trajectory** | **Male aged ≥ 65y** | | | | | | |
| **Stable healthy** | 1.00 | *Ref* | *Ref* |  | 1.00 | *Ref* | *Ref* |
| **Decreasing risk** | 1.01 | 0.05 | 65,983 |  | 1.01 | -0.026 | -0.081 |
| **Lower moderate risk** | 1.15*** | 0.26*** | 473,281*** |  | 1.16*** | 0.183*** | 1.745*** |
| **Higher moderate risk** | 1.19*** | 0.32*** | 617,134*** |  | 1.19*** | 0.273*** | 2.564*** |
| **Lower severe risk** | 1.12* | 0.14 | 267,937* |  | 1.13 | 0.181 | 1.583 |
| **Higher severe risk** | 1.16*** | 0.25** | 469,016** |  | 1.14* | 0.163 | 1.501 |
| **Health behavior trajectory** | **Female** | | | | | | |
| **Stable healthy** | 1.00 | *Ref* | *Ref* |  | 1.00 | *Ref* | *Ref* |
| **Decreasing risk** | 1.22*** | 0.25*** | 311,706*** |  | 1.22*** | 0.126*** | 1.264*** |
| **Lower moderate risk** | 1.10*** | 0.11*** | 125,256*** |  | 1.09*** | 0.054** | 0.516** |
| **Higher moderate risk** | 1.18*** | 0.24*** | 284,252*** |  | 1.18*** | 0.110*** | 1.042*** |
| **Lower severe risk** | 1.07** | 0.09** | 96,356** |  | 1.07** | 0.055** | 0.440** |
| **Higher severe risk** | 1.50*** | 0.56*** | 868,808*** |  | 1.50** | 0.374*** | 3.837** |
| **Health behavior trajectory** | **Female aged ≤ 64y** | | | | | | |
| **Stable healthy** | 1.00 | *Ref* | *Ref* |  | 1.00 | *Ref* | *Ref* |
| **Decreasing risk** | 1.28*** | 0.28*** | 208,922*** |  | 1.27*** | 0.190*** | 0.856*** |
| **Lower moderate risk** | 1.11*** | 0.11*** | 72,809*** |  | 1.11** | 0.049* | 0.264* |
| **Higher moderate risk** | 1.22*** | 0.26*** | 178,053*** |  | 1.22*** | 0.115*** | 0.596*** |
| **Lower severe risk** | 1.08** | 0.08** | 51,536** |  | 1.08** | 0.053* | 0.227** |
| **Higher severe risk** | 1.53*** | 0.62*** | 545,901*** |  | 1.52*** | 0.439*** | 2.153*** |
| **Health behavior trajectory** | **Female aged ≥ 65y** | | | | | | |
| **Stable healthy** | 1.00 | *Ref* | *Ref* |  | 1.00 | *Ref* | *Ref* |
| **Decreasing risk** | 1.14*** | 0.16*** | 302,516*** |  | 1.14 | -0.055 | 0.289 |
| **Lower moderate risk** | 1.08*** | 0.12*** | 200,786*** |  | 1.08 | 0.041 | 0.656 |
| **Higher moderate risk** | 1.16*** | 0.22*** | 405,659*** |  | 1.16** | 0.086** | 1.389** |
| **Lower severe risk** | 1.05** | 0.14** | 197,612** |  | 1.05** | 0.106* | 0.969* |
| **Higher severe risk** | 1.49*** | 0.48*** | 1,198,16*** |  | 1.49* | 0.301** | 5.117* |
| ***All covariates were controlled / InTME: Inpatient total medical expenditure / LOS: Length of stay / * P<0.05, ** P<0.01, *** P<0.001** | | | | | | | |
