# Supplementary material for: Joint trajectories of health behaviors and their associations with healthcare utilization and expenditure
Source: Int J Public Health. 2026 Sep 25;71:1609686. doi: 10.3389/ijph.2026.1609686 (PMC13669875; doi:10.3389/ijph.2026.1609686)
Supplement: Supplementary file 8 [file Table7.docx]

**Supplementary Table S7. Annual health behavior characteristics by trajectory group among females (South Korea, 2026)**

| **Types of trajectories** | **Categories** | **Year** | | | | **Trends** |
| --- | --- | --- | --- | --- | --- | --- |
|  |  | **2002-2003** | **2004-2005** | **2006-2007** | **2008-2009** |  |
| **Lower severe risk** | Tobacco use | Between 0 cigarettes  and 1-9 cigarettes per/day | Between 0 cigarettes  and 1-9 cigarettes per/day | Between 0 cigarettes  and 1-9 cigarettes per/day | Between 0 cigarettes  and 1-9 cigarettes per/day | Stable |
|  | Alcohol consumption | 1-9.9g per/day | 1-9.9g per/day | 1-9.9g per/day | 1-9.9g per/day | Stable |
|  | PA | 1-2 times per/week | 1-2 times per/week | 1-2 times per/week | 1-2 times per/week | Stable |
|  | BMI | 18.5-22.9kg/m^2^ | 18.5-22.9kg/m^2^ | 18.5-22.9kg/m^2^ | 18.5-22.9kg/m^2^ | Stable |
| **Stable healthy** | Tobacco use | 0 cigarettes per/day | 0 cigarettes per/day | 0 cigarettes per/day | 0 cigarettes per/day | Stable |
|  | Alcohol consumption | 0g per/day | 0g per/day | 0g per/day | 0g per/day | Stable |
|  | PA | Between 0 times and 1-2 times per/week | Between 0 times and 1-2 times per/week | 1-2 times per/week | 1-2 times per/week | Increasing |
|  | BMI | 18.5-22.9kg/m^2^ | 18.5-22.9kg/m^2^ | 18.5-22.9kg/m^2^ | 18.5-22.9kg/m^2^ | Stable |
| **Lower moderate risk** | Tobacco use | 0 cigarettes per/day | 0 cigarettes per/day | 0 cigarettes per/day | 0 cigarettes per/day | Stable |
|  | Alcohol consumption | 0g per/day | 0g per/day | 0g per/day | 0g per/day | Stable |
|  | PA | Between 0 times and 1-2 times per/week | 1-2 times per/week | 1-2 times per/week | 1-2 times per/week | Increasing |
|  | BMI | 23.0-24.9kg/m^2^ | 23.0-24.9kg/m^2^ | 23.0-24.9kg/m^2^ | 23.0-24.9kg/m^2^ | Stable |
| **Higher moderate risk** | Tobacco use | 0 cigarettes per/day | 0 cigarettes per/day | 0 cigarettes per/day | 0 cigarettes per/day | Stable |
|  | Alcohol consumption | 0g per/day | 0g per/day | 0g per/day | 0g per/day | Stable |
|  | PA | Between 0 times and 1-2 times per/week | 1-2 times per/week | 1-2 times per/week | 1-2 times per/week | Increasing |
|  | BMI | 25.0-29.9kg/m^2^ | 25.0-29.9kg/m^2^ | 25.0-29.9kg/m^2^ | 25.0-29.9kg/m^2^ | Stable |
| **Decreasing Risk** | Tobacco use | 0 cigarettes per/day | 0 cigarettes per/day | 0 cigarettes per/day | 0 cigarettes per/day | Stable |
|  | Alcohol consumption | 1-9.9g per/day | 1-9.9g per/day | Between 0g and  1-9.9g per/day | Between 0g and  1-9.9g per/day | Decreasing |
|  | PA | Between 0 times and 1-2 times per/week | 1-2 times per/week | 1-2 times per/week | 1-2 times per/week | Increasing |
|  | BMI | 25.0-29.9kg/m^2^ | Between 25.0-29.9kg/m2 and 23.0-24.9kg/m2 | Between 25.0-29.9kg/m2 and 23.0-24.9kg/m2 | Between 25.0-29.9kg/m2 and 23.0-24.9kg/m2 | Decreasing |
| **Higher severe risk** | Tobacco use | 1-9 cigarettes per/day | 1-9 cigarettes per/day | 1-9 cigarettes per/day | 1-9 cigarettes per/day | Stable |
|  | Alcohol consumption | 1-9.9g per/day | 1-9.9g per/day | 1-9.9g per/day | 1-9.9g per/day | Stable |
|  | PA | Between 0 times and 1-2 times per/week | Between 0 times and 1-2 times per/week | Between 0 times and 1-2 times per/week | Between 0 times and 1-2 times per/week | Stable |
|  | BMI | Between 23.0-24.9kg/m^2^ and 25.0-29.9kg/m^2^ | Between 23.0-24.9kg/m^2^ and 25.0-29.9kg/m^2^ | Between 23.0-24.9kg/m^2^ and 25.0-29.9kg/m^2^ | Between 23.0-24.9kg/m^2^ and 25.0-29.9kg/m^2^ | Stable |
