# Supplementary material for: Joint trajectories of health behaviors and their associations with healthcare utilization and expenditure
Source: Int J Public Health. 2026 Sep 25;71:1609686. doi: 10.3389/ijph.2026.1609686 (PMC13669875; doi:10.3389/ijph.2026.1609686)
Supplement: Supplementary file 9 [file Table8.docx]

| **Supplementary Table S8. Adjusted associations between smoking, alcohol consumption, and physical activity trajectories and healthcare outcomes by  sex, with BMI adjusted as a covariate (South Korea, 2026)** | | | | | | | | | | | | | | | | | | | | | | | |
| --- | --- | --- | --- | --- | --- | --- | --- | --- | --- | --- | --- | --- | --- | --- | --- | --- | --- | --- | --- | --- | --- | --- | --- |
| **Variables** | **OutTME** | | | | |  | **InTME** | | | | |  | **No. of outpatient visit** | | | | |  | **LOS** | | | | |
|  | ***B*** | **95% CI** | | | **P-value** |  | ***B*** | **95% CI** | | | **P-value** |  | ***B*** | **95% CI** | | | **P-value** |  | ***B*** | **95% CI** | | | **P-value** |
| **Health behavior trajectory** | **Male** | | | | | | | | | | | | | | | | | | | | | | |
| **Stable healthy** | *Ref* |  |  |  |  |  | *Ref* |  |  |  |  |  | *Ref* |  |  |  |  |  | *Ref* |  |  |  |  |
| **Decreasing risk** | **-0.116** | **-(0.18** | **-** | **-0.05)** | **0.000** |  | 0.069 | -(0.11 | - | 0.25) | 0.445 |  | **-0.063** | **-(0.100** | **-** | **-0.03)** | **0.000** |  | 0.077 | -(0.05 | - | 0.20) | 0.231 |
| **Lower moderate risk** | **0.054** | **(0.07** | **-** | **0.07)** | **<.0001** |  | **0.183** | **(0.13** | **-** | **0.23)** | **<.0001** |  | -0.008 | -(0.02 | - | 0.00) | 0.082 |  | **0.092** | **(0.05** | **-** | **0.13)** | **<.0001** |
| **Higher moderate risk** | -0.016 | -(0.03 | - | 0.00) | 0.096 |  | **0.269** | **(0.22** | **-** | **0.31)** | **<.0001** |  | **-0.042** | **-(0.05** | **-** | **-0.03)** | **<.0001** |  | **0.227** | **(0.19** | **-** | **0.27)** | **<.0001** |
| **Lower severe risk** | **0.082** | **(0.06** | **-** | **0.10)** | **<.0001** |  | **0.376** | **(0.32** | **-** | **0.43)** | **<.0001** |  | **0.030** | **(0.02** | **-** | **0.04)** | **<.0001** |  | **0.313** | **(0.18** | **-** | **0.44)** | **<.0001** |
| **Higher severe risk** | **0.126** | **(0.07** | **-** | **0.18)** | **<.0001** |  | **0.599** | **(0.39** | **-** | **0.81)** | **<.0001** |  | **0.029** | **(0.01** | **-** | **0.03)** | **0.016** |  | **0.219** | **(0.18** | **-** | **0.26)** | **<.0001** |
|  |  |  |  |  |  |  |  |  |  |  |  |  |  |  |  |  |  |  |  |  |  |  |  |
| **Health behavior trajectory** | **Female** | | | | | | | | | | | | | | | | | | | | | | |
| **Stable healthy** | *Ref* |  |  |  |  |  | Ref |  |  |  |  |  | *Ref* |  |  |  |  |  | *Ref* |  |  |  |  |
| **Decreasing risk** | **0.069** | **(0.04** | **-** | **0.09)** | **<.0001** |  | **0.294** | **(0.23** | **-** | **0.36)** | **<.0001** |  | **0.037** | **(0.02** | **-** | **0.05)** | **<.0001** |  | **0.189** | **(0.14** | **-** | **0.24)** | **<.0001** |
| **Lower moderate risk** | **0.050** | **(0.03** | **-** | **0.06)** | **<.0001** |  | **0.155** | **(0.11** | **-** | **0.20)** | **<.0001** |  | **0.043** | **(0.03** | **-** | **0.05)** | **<.0001** |  | **0.054** | **(0.01** | **-** | **0.04)** | **<.0001** |
| **Higher moderate risk** | **0.089** | **(0.07** | **-** | **0.11)** | **<.0001** |  | **0.286** | **(0.24** | **-** | **0.33)** | **<.0001** |  | **0.068** | **(0.06** | **-** | **0.08)** | **<.0001** |  | **0.176** | **(0.14** | **-** | **0.22)** | **<.0001** |
| **Lower severe risk** | -0.020 | -(0.04 | - | -0.01) | 0.354 |  | **0.101** | **(0.04** | **-** | **0.16)** | **0.002** |  | **-0.016** | **-(0.03** | **-** | **-0.00)** | **0.006** |  | **0.056** | **(0.01** | - | **0.10)** | **0.021** |
| **Higher Severe risk** | **0.157** | **(0.09** | **-** | **0.22)** | **<.0001** |  | **0.645** | **(0.51** | **-** | **0.78)** | **<.0001** |  | **0.063** | **(0.03** | **-** | **0.99)** | **0.000** |  | **0.489** | **(0.34** | **-** | **0.63)** | **<.0001** |

***All covariates were controlled for; BMI was additionally included as an adjustment variable / OutTME: Outpatient total medical expenditure / InTME: Inpatient total medical expenditure / LOS: Length of stay**
