# Supplementary material for: Joint trajectories of health behaviors and their associations with healthcare utilization and expenditure
Source: Int J Public Health. 2026 Sep 25;71:1609686. doi: 10.3389/ijph.2026.1609686 (PMC13669875; doi:10.3389/ijph.2026.1609686)
Supplement: Supplementary file 10 [file Table9.docx]

**Supplementary Table S9. Adjusted effect of health behavior trajectories on Out/InTME per visit by sex (South Korea, 2026)**

| **Variables** | **OutTME per visit** | | | | **InTME per visit** | | | |
| --- | --- | --- | --- | --- | --- | --- | --- | --- |
|  | ***B*** | **95% CI** | | **P-value** | ***B*** | **95% CI** | | **P-value** |
| **Health behavior trajectory** | **Male** | | | | | | | |
| **Stable healthy** | *Ref* |  |  |  | *Ref* |  |  |  |
| **Decreasing risk** | -0.006 | (-0.02 | 0.01) | 0.323 | **0.026** | **(0.01** | **0.04)** | **0.000** |
| **Lower moderate risk** | 0.005 | (-0.01 | 0.02) | 0.418 | 0.041 | (0.00 | 0.09) | 0.058 |
| **Higher moderate risk** | **-0.031** | **(-0.04** | **-0.02)** | **<.0001** | **0.049** | **(0.03** | **0.07)** | **<.0001** |
| **Lower severe risk** | **0.017** | **(0.01** | **0.03)** | **0.017** | **0.058** | **(0.04** | **0.07)** | **<.0001** |
| **Higher severe risk** | **0.046** | **(0.02** | **0.06)** | **<.0001** | **0.054** | **(0.01** | **0.12)** | **0.049** |
| **Health behavior trajectory** | **Female** | | | | | | | |
| **Stable healthy** | *Ref* |  |  |  | *Ref* |  |  |  |
| **Decreasing risk** | **0.018** | **(0.00** | **0.03)** | **0.018** | **0.046** | **(0.02** | **0.07)** | **<.0001** |
| **Lower moderate risk** | **0.017** | **(0.01** | **0.03)** | **0.000** | 0.001 | (-0.02 | 0.02) | 0.854 |
| **Higher moderate risk** | **0.018** | **(0.01** | **0.03)** | **0.001** | 0.000 | (-0.02 | 0.02) | 0.977 |
| **Lower severe risk** | -0.012 | (-0.03 | 0.00) | 0.069 | **0.041** | **(0.02** | **0.06)** | **<.0001** |
| **Higher Severe risk** | **0.032** | **(0.02** | **0.04)** | **<.0001** | **0.077** | **(0.02** | **0.14)** | **0.011** |

***All covariates were controlled / OutTME: Outpatient total medical expenditure / InTME: Inpatient total medical expenditure**
